# The Regulation of Ant Colony Foraging Activity without Spatial Information

**DOI:** 10.1371/journal.pcbi.1002670

**Published:** 2012-08-23

**Authors:** Balaji Prabhakar, Katherine N. Dektar, Deborah M. Gordon

**Affiliations:** 1 Departments of Computer Science and Electrical Engineering, Stanford University, Stanford, California, United States of America; 2 Biomedical Computation, School of Engineering, Stanford University, Stanford, California, United States of America; 3 Department of Biology, Stanford University, Stanford, California, United States of America; Princeton University, United States of America

## Abstract

Many dynamical networks, such as the ones that produce the collective behavior of social insects, operate without any central control, instead arising from local interactions among individuals. A well-studied example is the formation of recruitment trails in ant colonies, but many ant species do not use pheromone trails. We present a model of the regulation of foraging by harvester ant (*Pogonomyrmex barbatus*) colonies. This species forages for scattered seeds that one ant can retrieve on its own, so there is no need for spatial information such as pheromone trails that lead ants to specific locations. Previous work shows that colony foraging activity, the rate at which ants go out to search individually for seeds, is regulated in response to current food availability throughout the colony's foraging area. Ants use the rate of brief antennal contacts inside the nest between foragers returning with food and outgoing foragers available to leave the nest on the next foraging trip. Here we present a feedback-based algorithm that captures the main features of data from field experiments in which the rate of returning foragers was manipulated. The algorithm draws on our finding that the distribution of intervals between successive ants returning to the nest is a Poisson process. We fitted the parameter that estimates the effect of each returning forager on the rate at which outgoing foragers leave the nest. We found that correlations between observed rates of returning foragers and simulated rates of outgoing foragers, using our model, were similar to those in the data. Our simple stochastic model shows how the regulation of ant colony foraging can operate without spatial information, describing a process at the level of individual ants that predicts the overall foraging activity of the colony.

## Author Summary

Social insect colonies operate without any central control. Their collective behavior arises from local interactions among individuals. Here we present a simple stochastic model of the regulation of foraging by harvester ant (*Pogonomyrmex barbatus*) colonies, which forage for scattered seeds that one ant can retrieve on its own, so there is no need for pheromone trails to specific locations. Previous work shows that colony foraging activity is regulated in response to current food availability, using the rate of brief antennal contacts inside the nest between foragers returning with food and outgoing foragers. Our feedback-based algorithm estimates the effect of each returning forager on the rate at which foragers leave the nest. The model shows how the regulation of ant colony foraging can operate without spatial information, describing a process at the level of individual ants that predicts the overall foraging activity of the colony.

## Introduction

The fundamental question about the collective behavior of animals is how the actions of individuals add up to the dynamic behavior we observe. In many systems, including animal groups, distributed networks are regulated using feedback based on local interactions. It is not yet clear how the analogies among diverse complex systems reveal general underlying processes [1], [2]. Here we propose a simple stochastic model of collective behavior in ants. Our first goal is to account for the details of a particular system, as a step toward further insight on whether similar processes are at work in othersystems. A second goal of our work is to contribute to the study of collective behavior from the perspective of evolutionary biology. If the outcome of collective behavior is ecologically important, then natural selection can act on variation in that behavior. Modeling the parameters that produce collective behavior can provide the basis for detailed measures of variation among ant colonies.

The best-studied algorithms for collective behavior in animals are those that regulate spatial patterns [3], based on local interactions that influence whether one animal stays close to another [4]. Social insect colonies provide many fascinating examples of collective behavior. There is no central control; no insect directs the behavior of another. Like other social insects, ants use local interactions to regulate colony behavior [5].

The most familiar example of feedback based on local interaction in ants is recruitment to a food source using a pheromone trail. In some ant species, an ant that finds food lays a chemical trail on its way back to the nest. Studies of the algorithms used by ants in forming recruitment trails show that a slight tendency on the part of other ants to move toward the trail leads to the formation of trail systems [6], [7], [8] that can channel ants to the best food source [9] or trace the shortest path toward the food [10]. Ants also use feedback from other forms of interaction, such as brief antennal contact, in recruitment to food [11] and in other spatial decisions. The perception of the local density of digging ants generates branches in nest chambers [12]. The rate of brief antennal contact as a cue to local density [13] is used in spatial decisions, in combination with other information about location, such as the choice of new nest sites by acorn ants [14], [15].

The regulation of activity by a simple stochastic process is characteristic of many biological systems. Local interactions in social insects, like those in other dynamical networks, can regulate the flow or intensity of activity as well as its location or spatial pattern. For example, a social insect colony must adjust the allocation of individuals to various tasks, in response to changing conditions [16]. Various models have been proposed to explain the dynamics of the intensity of activity, or numbers of workers, devoted to colony tasks [e.g. 17], [18], [19].

Here we present a simple stochastic model that explains the process underlying the regulation of foraging activity in harvester ant (*Pogonomyrmex barbatus*) colonies. Foraging activity, the numbers of ants currently foraging, changes from moment to moment within a foraging period and from day to day. This species does not use pheromone trails to recruit to localized food sources. The ants forage for seeds that are scattered by wind and flooding [20], not distributed in patches, and a single ant can retrieve a seed on its own. The model uses an algorithm based on local interactions among individuals in the form of brief antennal contacts, without any spatial information such as pheromone trails.

Harvester ants searching for food in the desert undergo desiccation, and the ants obtain water from metabolizing the fats in the seeds they eat. Thus a colony must spend water to obtain water, as well as food. The intensity of foraging is regulated from moment to moment, and from day to day, to adjust foraging activity to current food availability, while maintaining sufficient numbers of ants foraging to compete with neighbors for foraging area [21].

A long-term study of the foraging ecology of this species has shown how the moment-to-moment regulation of foraging is accomplished. Regulation depends on feedback from returning foragers, who stimulate the outgoing foragers to leave on the next trip. Forager return rate corresponds to food availability, because foragers almost always continue to search until they find a seed, then immediately bring it back to the nest [22], [23]. The more food is available, the less time foragers spend searching and the more rapidly they return to the nest.

The crucial interactions between returning and outgong foragers take place in a narrow entrance tunnel, 5–10 cm long, that leads to a deeper entrance chamber. Observations with a videoscope show that returning foragers drop their seeds in the tunnel, and then other ants pick up the seeds and take them deeper into the nest. Once the returning forager has dropped its seed, it becomes an outgoing forager, available to go out on its next trip. Experiments using artificial ant mimics coated with extracts of ant cuticular hydrocarbons [24], [25], and experiments manipulating the rate of forager return [26], [27], [28], [29] show that how quickly an outgoing forager leaves on its next trip depends on its interactions with returning foragers. Foraging activity is more closely regulated when foraging rates are high, above a baseline rate at which foragers leave independently of the rate of forager return [29].

We developed a model that takes into account previous work on the regulation of foraging. We compared simulations using the model with new data, from field experiments, that show how the rate of at which outgoing foragers leave the nest changes in response to changes in the rate at which returning foragers go back to the nest.

## Methods

### Measures of harvester ant foraging activity

Experiments manipulating forager return rate were performed in August 2009 and August–September 2010 at the site of a long-term study since 1985 of a population of *P. barbatus* near Rodeo, New Mexico, USA. In 2009 there were 33 trials in 9 colonies on 8 days, and in 2010 there were 29 trials in 8 colonies on 5 days, of which 4 were the same colonies as in 2009, for a total of 62 trials. All colonies were mature, more than 5 years old (ages determined by yearly census; methods in [21]).

Returning foragers were prevented from returning from the nest in minutes 4–7 of a 20-min observation; methods were the same as in [28], [29]. Rates of returning and outgoing foragers crossing an imaginary line along the trail were measured from video film using an image analysis system developed by Martin Stumpe (http://www.antracks.org). This image analysis system made it possible to measure foraging rates accurately on a shorter timescale than in previous work.

Most colonies use more than one foraging direction on a given day [21]. We filmed all trails and used the combined foraging rates for all trails. Foraging rates were calculated separately for the periods before (0–240 sec), during (240–430 sec), and after (500–1100 sec) the removal of returning foragers, as in previous work [28], [29]. The small interval between 430 and 500 sec allows for the time it takes ants passing the camera on the foraging trail to reach the nest. To correct for differences among trails in the distance between the camera and the nest, we found the average time, out of 5 observations, for an ant to travel back to the nest from the point where the foraging trail was filmed. To adjust for the distance between the camera and the nest, we then subtracted this travel time from the counts for outgoing foragers and added it to counts for returning foragers.

The average error in the accuracy of the image analysis software in counting foraging rate was 7.3%, estimated by comparing 66 counts made by observers from 500 frames (about 17 sec) of 44 video films with counts made by the image analysis software. Most errors were due to an extraneous object or shadow in the film at the point at which ants crossed the imaginary line where ants were counted. There was no bias toward counting more or fewer ants than actually crossed the line.

## Results

### Model of the regulation of foraging activity

**Figure 1:**
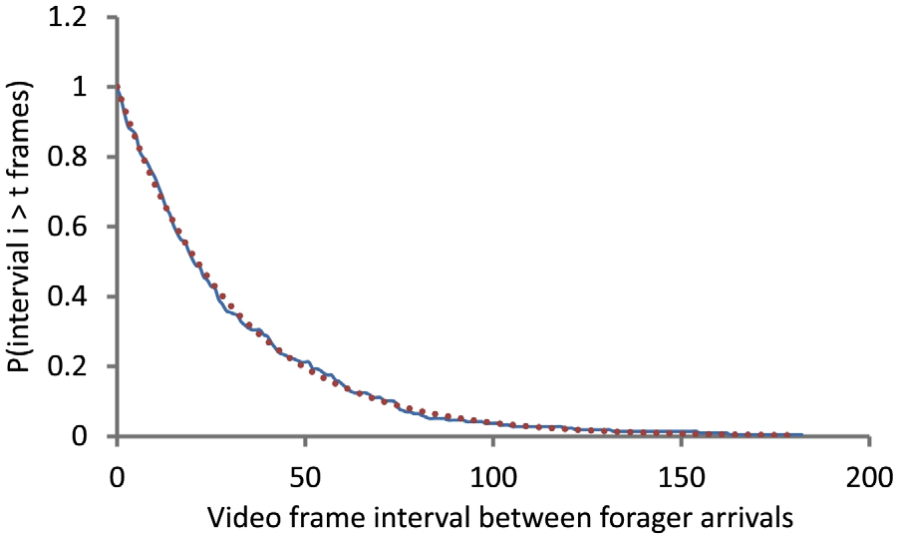
Poisson distribution of intervals between the arrival of successive returning foragers at the nest. The figure shows representative data from one trial in which returning foragers were removed. Intervals are shown in video frames; each frame is 1/30 sec. The y-axis is the probability that the interval *i* between successive returning foragers exceeds *t* frames. The solid blue line corresponds to the real data and the dotted red line corresponds to the exponential fit y = *e* ^−0.0326^ *t* with a mean separation time of 1/0.0326 or 30.67 frames, equal to 1.02 seconds.

We first tested whether the return of foragers to the nest could be described as a Poisson process. To determine the distribution of intervals between the arrival of foragers at the nest, we used the data from the period before the removal of returning foragers (60–240 sec) in 39 trials. We calculated the fit with an exponential distribution of the empirical distribution of the interarrival times of the returning foragers, and measured the error using the total variation distance [30] between the two distributions. We found that the return of foragers to the nest can be described as a Poisson process: the distribution of intervals between returning foragers is exponential (Fig. 1) and independent of the spacing between foragers in the previous interval. We found the mean (SE) error for the 39 trials to be 0.056 (0.009) with a better fit at high foraging rates.

We began with a simple linear model in which the rate of outgoing foragers *x(t)* depends on the the rate of returning foragers plus a constant base rate:

(1) *λ(t) = λ_ac_ f(t,τ)*
(2) *x(t) = Poisson (λ_b_)*+*Poisson (λ (t))*

where *λ_b_* is a baseline rate of outgoing foragers, independent of the rate at which other foragers return; *λ_ac_* sets the number of outgoing foragers per returning forager; and *f(t, τ)* is the number of returning foragers between times *t*−*τ* and *t*.

For this initial, linear model, the most important parameter in predicting the rate of outgoing foragers is *τ*. Because this model integrates over all returns in time *τ*, fitting the model requires us to choose a value of *t* that gives an equally good fit to the observed data over a range of foraging rates. This is difficult because foraging rates vary greatly among colonies, days, and moment-to-moment changes in the conditions that foragers encounter [5], [27], [29]. We thus elaborated this model into a second one that avoids the integration of instantaneous arrivals over a time interval that is not uniform across different foraging conditions. Moreover, the model below captures the effect of a single returning forager and so explains the process at the level of individual ants, as for example in other non-linear models that describe pheromone trail foraging by ants [7].

The model operates in discrete time: returning foragers are observed in successive and equal time slots. We denote the rate of outgoing foragers as ‘α’, which increases by an amount *c*>0 for each returning food-bearing forager. Alpha decreases by an amount *q*>0 for each forager that leaves the nest, because the departure of each outgoing forager decreases the number of outgoing foragers in the queue at the nest entrance available to meet returning foragers. Alpha decays by an amount *d*>0 during each time slot, to reflect the lack of response to very low interaction rate [22]. Finally, α has a lower bound, α., to reflect the observation that outgoing foragers leave the nest at a fixed low rate even when no foragers return for a while [28], [29].

We assume that arrivals occur at the beginning of time slots and departures occur at the end of time slots. For *n* = 1, 2, …, let *A_n_* denote the number of returning food-bearing foragers in the *n*th time slot, and let *D_n_* denote the number of outgoing foragers leaving the nest. The rate at which ants leave the nest in the *n^th^* slot is α*_n_*, *n* = 1, 2, …. We assume that α*_n_*≥α>0 for *n* = 1, 2, …, where α is a parameter. The number of departures at the end of the *n^th^* time slot, *D_n_*, was set equal to a Poisson random variable of mean α*_n_*. Given the α*_n_*, the *D_n_* are statistically independent of each other and of *A_n_*. The dynamics of α*_n_* are described by:

(3) α*_n_* = max (α*_n_*_−1_−*qD_n_*_−1_+*cA_n_−d*, α), α_0_ = 0
(4) *D_n_*∼*Poisson* (α*_n_*)

**Figure 2:**
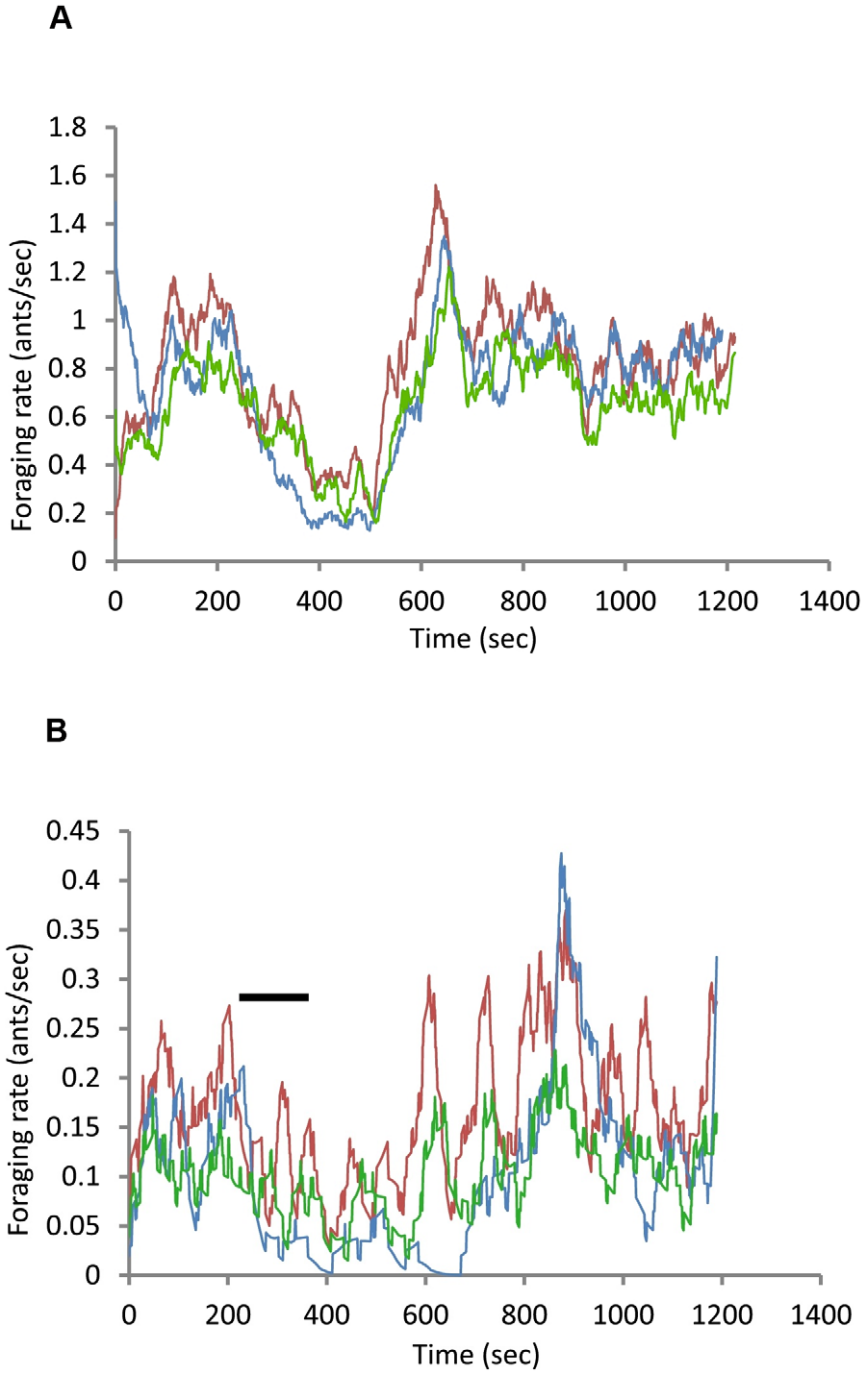
Comparison of observed and simulated foraging rates. The rate of returning foragers was experimentally decreased by removing the returning foragers, leading to a decrease in the rate at which outgoing foragers left the nest. Each figure shows data from one trial. Returning foragers were removed from 240–420 sec, during the interval indicated by the horizontal black line, and then allowed to return to the nest undisturbed for the remainder of the trial. The red line shows the observed rate of returning foragers, the blue line shows the observed rate of outgoing foragers, and the green line shows the simulated rate of outgoing foragers. a, high foraging rate (mean rate returning foragers 0.807 ants/sec); b, low foraging rate (mean rate returning foragers 0.169 ants/sec).

In developing the model, we sought to facilitate its future use to examine variation among colonies within this species, for example in the baseline rate at which ants leave the nest even when no ants return [29], and to take into account three features of the observed behavior of the ants in response to experimental manipulation of foraging rate [28], [29]. First, there is a lag in the recovery of the rate of outgoing foragers in response to recovery of the rate of returning foragers after a decline [28], [29] (Fig. 2). Second, we introduced *q*, the extent to which each departing forager empties the nest entrance of available foragers, because observations with a videoscope inside the nest show that outgoing ants are crowded in a small tunnel, so that once each outgoing ant departs it takes some time for the next outgoing ant to move to the top of the tunnel where it can meet returning foragers. Third, we include the decay parameter *d* because experimental results show that the response to returning ants is weaker after some time has elapsed since the last ant returned [24].

### Comparison of model and data

We compared the simulated output of the model with the data from field experiments on the response of outgoing foragers to a range of rates of returning foragers (Fig. 2). Using as input the data on the rate of returning foragers, we generated the simulated rate of outgoing foragers, adjusting one parameter and evaluating the resulting match with the observed rate of outgoing foragers.

The model has four parameters: α, *c*, *q* and *d*. We examined the fit between model and data for one parameter, *c*. We thus fixed α., *q* and *d* and varied *c*. As with any birth-death process, the ratio of *c* to *e* determines the distribution of {α*_n_*}. We set *q* to 0.05 to keep the range of values of α*_n_* within the range of observed foraging rates (0.15 to 1.2 ants per sec). We set *d* to 0 for the simulations reported here; however, empirical studies show that *d* may be an important parameter because it may vary by colony [29], or in response to variation in environmental conditions that could affect the rate of decay of chemical cues such as the cuticular hydrocarbons that ants assess by antennal contact [24]. Similarly, α, the baserate of foraging, was very small, equal to 0.01 ants per second [27].

To choose *c* for the given values of α, *q*, and *d*, we used for each of the 62 experimental trials the data on returning foragers and equations (3) and (4) to generate a simulated rate of outgoing foragers. We swept across values of *c* from 0.01 to 0.25, and found the relative root-mean-square error (RMSE) [31] between the simulated and observed rates of outgoing foragers in each time interval for each of 200 iterations. Each iteration produces a different output trace because of the independent Poisson random variable generated at equation (4). We chose the *c* with the lowest average RMSE, over the 200 iterations, between simulated and observed rates of outgoing foragers. The RMSE for the best *c* for each trial ranged from 0.237 to 4.9, and the mean (SE) RMSE for the 62 values chosen was 0.602 (0.077).

To evaluate how well our estimate of *c* captured, for a given colony, the effect of each returning forager on the rate of outgoing foragers, we compared the error among runs of the simulation, due to randomness in the departures at equation (4), with the error produced by varying *c*. To do this we compared the RMSE between observed and simulated rates of outgoing foragers among simulated runs with the RMSE for different values of *c* among trials of the same colony. We first found for each trial the mean range in RMSE, between observed and simulated rates of outgoing foragers, among 200 iterations of the simulation using the same value of *c*. To estimate the difference among runs of the simulation, we used each trace of returning foragers to produce 3 different traces of simulated outgoing foragers (A,B,C). We found the RMSE for A vs B and A vs C, and repeated this 200 times per trial at foraging rates ranging, as did the observed rates, from low (0.1 returning ants/sec) to high (1.2 returning ants/sec). We then found the mean RMSE when varying values of the best *c* among trials for the same colony. To do this we chose arbitrarily, for each of the 13 colonies, 2 of the 3 or 4 trials. For those 2 trials we found the average of the values of *c*, then found the RMSE as above [31] for simulated values of rates of outgoing foragers for each of the 1 or 2 remaining trials for that colony in the same year. We deleted one of the remaining trials in cases when foraging rates were much lower than for other trials with the same colony.

The results of this comparison indicate that our estimate of best *c* generates the observed outgoing forager rate within the same range of error as the error generated by randomness in forager departures. The mean (SE) change in RMSE among simulated runs was 13% (0.0495) at low foraging rates and 2.6% (0.01) at high foraging rates. The mean (SE) change in RMSE obtained by varying *u* among trials of the same colony was 15.5% (0.019).

**Figure 3:**
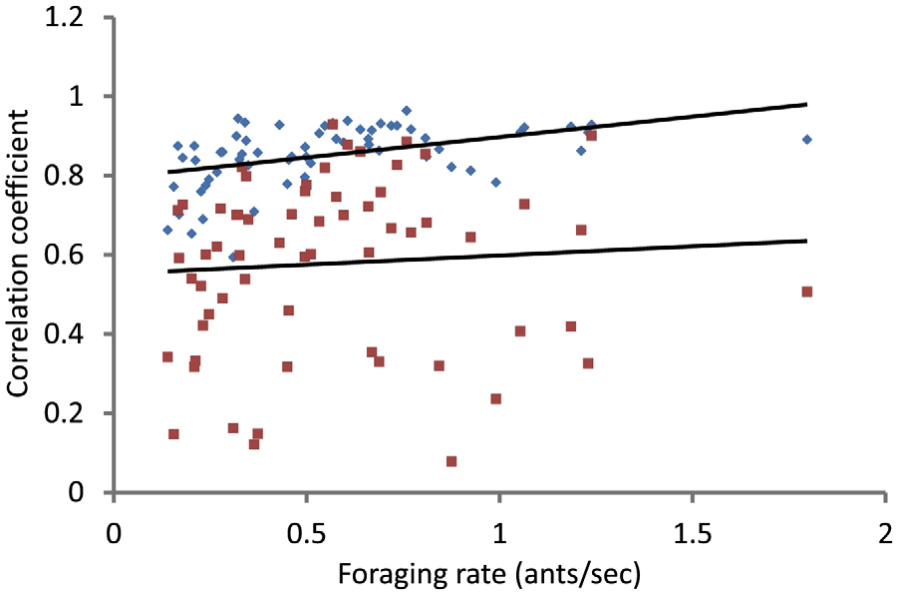
Correlation of rates of outgoing and returning foragers as a function of level of foraging activity. Each point shows, for one trial, the coefficient of correlation between the smoothed rates of returning and outgoing foragers. The x-axis shows the mean rate of returning foragers over the entire trial. Blue diamonds show the coefficients of correlation between observed rates of returning foragers and simulated rates of outgoing foragers. Red squares show the coefficients of correlation between observed rates of returning foragers and observed rates of outgoing foragers. The upper line shows the least-squares fit to the increase with foraging activity of the correlation between observed returning and simulated outgoing foraging rates points (blue); the lower line shows the least-squares fit to the increase with foraging activity of the correlation between observed returning and observed outgoing foraging rates(red).

We examined whether the correlation between rates of returning and outgoing foragers was at least as high in the simulation as in the data. To do this we compared the correlation between observed rates of returning and simulated rates of outgoing foragers, using the best value of *c*, with those between observed rates of returning and observed rates of outgoing foragers. We calculated the correlation between the observed rates of returning foragers and the simulated rate of outgoing foragers, in all 62 trials, by applying a moving-average rect filter [32] with a radius of 25 time slots, and then finding the empirical correlation coefficent between the smoothed traces [31]. We calculated in the same way the correlation between observed rates of returning and outgoing foragers. The simulated rates of outgoing foragers led to correlation coefficients higher than those between observed rates of returning and outgoing foragers (t-test, t = 8.98, p<0.001; Fig. 3).

Because previous work showed that the rate of outgoing foragers tracks the rate of forager return more closely when foraging rates are high, we examined how the correlation of rates of returning and outgoing foragers varies with foraging rate. The magnitude of the correlation coefficient between observed returning and simulated outgoing foragers increased with the mean rate of returning foragers (Spearmann's rank correlation, n = 62, z = −4.04, p = 0.0001, blue points in Fig. 3). The magnitude of the correlation coefficient for the observed returning and outgoing rates did not increase significantly with the mean rate of returning foragers (Spearmann's rank correlation, n = 62, z = −1.34, p = 0.18, red points in Fig. 3).

## Discussion

Our model provides a way to evaluate quantitatively the response of individuals to local interactions. It explains how ant colonies regulate foraging activity from moment to moment, in response to current food availability, without any central control or any spatial information on the location of food. Changes in a colony's foraging activity from moment to moment, and from day to day, show a predictable response to changes in forager return rate, despite considerable stochasticity (Fig. 2). Our results show that much of these changes in colony foraging activity can be explained by the effect of each returning forager on the probability that outgoing foragers leave the nest to search for food.

The model is analyzable because the distribution of returning foragers is well-approximated by a Poisson process at high foraging rates. Like cars on highways, returning foragers travel at different velocities and overtake each other; as [33] shows, this produces a Poisson process.

The simulated data provided by our model capture many of the features of a rich body of empirical results from a long-term study of the foraging behavior of harvester ant colonies. The model provides a simulated rate of outgoing foragers, in response to the rate of returning foragers, that is reasonably similar to the observed rate (Fig. 2), producing a close correlation between the rates of returning and outgoing foragers.

Another similarity between the model and observation is in the contrast between colony behavior when food availability is high, so that foragers find food quickly and the rate of forager return is high, and colony behavior when food availability is low, so that foragers find food slowly and the rate of forager return is low. Previous work on harvester ant foraging showed that rates of outgoing foragers are more closely adjusted to rates of returning foragers when foraging rates are high [29]. The same was true of our model. For example, Fig. 2 shows the data for two representative cases. When foraging rates are high (Fig. 2A), on a day when food availability is high and foragers find it quickly, the rate of returning and outgoing foragers is more closely matched than when foraging rates are low (Fig. 2B), so that foragers find food more slowly and return less frequently. The closer fit between outgoing and returning foragers at high foraging rates occurs because the range of values of α*_n_* tends to be much smaller and closer to α at low foraging rates than at high foraging rates, and this causes *D_n_* to be close to zero and, hence, less correlated with *A_n_*.

However, the simulation generally produces a closer correlation between the rates of returning and outgoing foragers than is observed in the data. The correlation coefficients for the observed rate of returning foragers and the simulated rate of outgoing foragers are higher than those for the observed rate of returning foragers with the observed rate of outgoing foragers (Fig. 3). In addition, the coefficient of correlation with the observed rate of returning foragers increased significantly with foraging rate for the simulated rate of outgoing foragers but does not increase significantly for the observed rate of outgoing foragers (Fig. 3).

The higher correlation in the simulation than in the data occurs because our model (equations 3 and 4) does not capture all of the factors that produce the actual rate at which ants leave the nest. For example, the structure of each nest probably affects the flow of ants in and out of the nest entrance, which in turn may affect the rate of interaction between outgoing and returning ants. A question for future work is whether nonlinear effects of nest structure influence the relation between overall foraging rate and the correlation of the rates of returning and outgoing foragers. Our model assumes the same relation between the rate of incoming and rate of outgoing foragers for all nests, and thus does not take into account the local influence of nest structure. Weather conditions also influence foraging activity, leading to day-to-day fluctuations in the foraging activity of a given colony [28].

The process described here is analogous to those operating in many other distributed networks, from computer networks to neural integrators, that regulate activity through the rate of interaction [34], [35]. Further work is needed to determine the details of the correspondence among these analogous systems; for example, in this system, the Poisson distribution of returning foragers is crucial.

The model presented here contributes to the study of the evolution of collective behavior in harvester ants, because it can be used to guide empirical measurement of differences among colonies in the regulation of foraging [29], by examining whether colonies tend to show characteristic parameter values. Harvester ant colonies differ in foraging behavior, and such differences persist from year to year as the colony grows older [21], [29]. Heritable variation among colonies in ecological relations, such as the regulation of foraging, is the source of variation in fitness [36]. Future work will examine differences among colonies in the response to interactions of returning and outgoing foragers. Small differences in the ants' response to local interactions may lead to ecologically important differences among colonies that shape the evolution of collective behavior.

## References

[1] Camazine S, Deneubourg J-L, Franks N R, Sneyd J, Theraulaz G, et al. (2003) Self-Organization in biological systems. Princeton: Princeton University Press. 529 p.

[2] Gordon DM (2007) Control without hierarchy. Nature 4468: 143. 10.1038/446143a 17344838

[3] Sumpter, D.J T (2010) Collective animal behavior. Princeton: Princeton University Press. 298 p.

[4] Torney C, Neufeld., Couzin ID (2009) Context-dependent interaction leads to emergent search behavior in social aggregates. Proc Natl Acad Sci USA 52: 22055–2206. 10.1073/pnas.0907929106 PMC2799714 20018696

[5] Gordon DM (2010) Ant encounters: interaction networks and colony behavior. Primers in complex systems. Princeton: Princeton University Press. 184 p.

[6] Detrain C, Deneubourg J-L (2008) Collective decision-making and foraging patterns in ants and honeybees. Adv In Insect Phys 35: 123–173.

[7] Sumpter DJT, Pratt SC (2003) A modelling framework for understanding social insect foraging. Behav Ecol Sociobiol 53: 131–144.

[8] Sumpter DJT, Beekman M (2003) From nonlinearity to optimality: pheromone trail foraging by ants. Anim Behav 66: 273–280.

[9] Beckers RJL, Deneubourg JL, Goss S, PasteelsJ M (1990) Collective decision-making through food recruitment. Insect Soc 37: 258–267.

[10] Dorigo M, Di Caro G, Gambardella LM (1999) Ant algorithms for discrete optimization. Artif Life 5: 137–172. 10633574 10.1162/106454699568728

[11] Mailleux AC, Buffin A, Detrain C, Deneubourg J-L (2010) Recruiter or recruit: who boosts the recruitment in starved nests in mass foraging ants? Anim Behav 79: 31–35.

[12] Toffin E, Di Paolo D, Campo A, Detrain C, Deneubourg J-L (2009) Shape transition during nest digging in ants. Procs Natl Acad Sci U S A 106: 18816–18620. 10.1073/pnas.0902685106 PMC2773997 19846774

[13] Gordon DM, Paul REH, Thorpe K (1993) What is the function of encounter patterns in ant colonies? Anim Behav 45: 1083–1100.

[14] Pratt S (2005) Quorum sensing by encounter rates in the ant *Temnothorax albipennis* . Behav Ecol 16: 488–496.

[15] Pratt S, Sumpter D (2006) A tunable algorithm for collective decision-making. Proc Nat Acad Sci U S A 103: 15906–15910. 10.1073/pnas.0604801103 PMC1635101 17038502

[16] Gordon DM (1996) The organization of work in social insect colonies. Nature 380: 121–124.

[17] Pacala SW, Gordon DM, Godfray HCJ (1996) Effects of social group size on information transfer and task allocation. Evol Ecol 10: 127–165.

[18] Anderson C, Ratnieks F (1999) Task partitioning in insect colonies. 1. Effect of colony size on queuing delay and colony ergonomic efficiency. Am Nat 154: 521–535. 10561125 10.1086/303255

[19] Karsai I, Schmikl T (2011) Regulation of task partitioning by a “common stomach”: a model of nest construction in social wasps. Behav Ecol 22: 819–830.

[20] Gordon DM (1993) The spatial scale of seed collection by harvester ants. Oecologia 95: 479–487. 10.1007/BF00317431 28313287

[21] Gordon DM, Kulig AW (1996) Founding, foraging and fighting: colony size and the spatial distribution of harvester ant nests. Ecol 77: 2393–2409.

[22] Gordon DM (1991) Behavioral flexibility and the foraging ecology of seed-eating ants. Am Nat 138: 379–411.

[23] Beverly B, McLendon H, Nacu S, Holmes S, Gordon DM (2009) How site fidelity leads to individual differences in the foraging activity of harvester ants. Behav Ecol 20: 633–638.

[24] Greene MJ, Gordon DM (2003) Cuticular hydrocarbons inform task decisions. Nature 423: 32. 12721617 10.1038/423032a

[25] Greene MJ, Gordon DM (2007) Interaction rate informs harvester ant task decisions. Behav Ecol 18: 451–455.

[26] Gordon DM (2002) The regulation of foraging activity in red harvester ant colonies. Am Nat 159: 509–518. 18707433 10.1086/339461

[27] Schafer RJ, Holmes S, Gordon DM (2006) Forager activation and food availability in harvester ants. Anim Behav 71: 815–822. 10.1016/j.anbehav.2013.05.012 PMC3767282 24031094

[28] Gordon DM, Holmes S, Nacu S (2008) The short-term regulation of foraging inharvester ants. Behav Ecol 19: 217–222.

[29] Gordon DM, Guetz A, Greene MJ, Holmes S (2011) Colony variation in the collective regulation of foraging by harvester ants. Behav Ecol 22: 429–435. 22479133 10.1093/beheco/arq218 PMC3071749

[30] Durrett R (1991) Probability: Theory and Examples. Cambridge Series in Statistical and Probabilistic Mathematics. New York: Cambridge University Press.

[31] Papoulis A (1965) Probability, Random Variables, and Stochastic Processes. New York: McGraw-Hill. 841 p.

[32] Foley J, van Dam A, Feiner S, Hughes J (1995) Computer Graphics: Principles and Practice, 2^nd^ Edition. Reading: C. Addison-Wesle. 1175 p.

[33] Breiman L (1963) The Poisson tendency in traffic distribution. Annals Math Stat 34: 308–311.

[34] Alizadeh M, Atikoglu B, Kabbani A, Lakshmikantha A, Pan R, et al. (2008) Data center transport mechanisms: congestion control theory and IEEE standardization. In: Proceedings of the 46th Annual Allerton Conference on Communications, Control and Computing.

[35] Goldman MS, Compte A, Wang X-J (2009) Neural integrator models. In: Encyclopedia of Neuroscience 6. Squire LR, editor. Oxford: Academic Press. pp. 165–178.

[36] Dingemanse NJ, Kazem AJN, Reale D, Wright J (2010) Behavioural reaction norms: animal personality meets individual plasticity. Trends Ecol Evol 25: 81–90. 19748700 10.1016/j.tree.2009.07.013

